# Complete genome sequence of an *Achromobacter xylosoxidans* strain H1_3_1 isolated from a hybrid biological-inorganic system reactor

**DOI:** 10.1128/MRA.00612-23

**Published:** 2023-10-27

**Authors:** Xiang Feng, Daichi Kazama, Kozo Sato, Hajime Kobayashi

**Affiliations:** 1 Department of Systems Innovation, Graduate School of Engineering, The University of Tokyo, Tokyo, Japan; 2 Frontier Research Center for Energy and Resources (FRCER), Graduate School of Engineering, The University of Tokyo, Tokyo, Japan; Montana State University, Bozeman, Montana, USA

**Keywords:** *Achromobacter*, hydrogen-oxidizing bacteria, chemolithoautotrophy, hybrid biological-inorganic system

## Abstract

We report the complete genome of *Achromobacter xylosoxidans* strain H1_3_1, which was isolated from a reactor of a hybrid biological-inorganic system. The complete genome comprised 7,071,873 bp, including 6,428 codings, 10 rRNA, and 70 tRNA, with 67.4% G + C content.

## ANNOUNCEMENT

*Achromobacter xylosoxidans* is a common bacterium causing respiratory infection in cystic fibrosis patients (1). Members of the genus *Achromobacter* are aerobic Gram-negative bacteria, which can utilize a variety of organic acids and amino acids as carbon sources (2). *A. xylosoxidans* strain H1_3_1 was isolated from an enrichment of halotolerant hydrogen-oxidizing bacteria. A brackish water sample from Shiokawa (26.66 N, 127.99 E), a brackish river in Okinawa, Japan, was inoculated into a hybrid biological-inorganic system reactor with the minimum medium described in reference 3 containing 180-mM phosphate buffer. After three subculturing in the reactors, the medium of the third reactor was spread onto the same medium solidified with 1% (wt/vol) gellan gum and cultured under H_2_/CO_2_/O_2_ (80:10:10) at 30°C. Colonies were further purified by streaking, resulting in the isolation of the strain H1_3_1. The strain H1_3_1 can grow chemolithoautotrophically under H_2_/O_2_/CO_2_ atmospheres. To better understand the genetic features leading to chemolithoautotrophy, the complete genome of *A. xylosoxidans* strain H1_3_1 was determined.

The strain H1_3_1 was propagated in 20 mL of the minimal medium under H_2_/CO_2_/O_2_ (80:10:10) at 30°C for 2 days and was harvested by centrifugation. Genomic DNA was extracted from the cell pellet using the Genomic-tip 20 /G Kit (Qiagen) with the Genomic DNA Buffer Set (Qiagen) according to the manufacturer’s protocol and was qualitatively analyzed using a 5,200-fragment analyzer system with an Agilent high-sensitivity genomic DNA 50-kb kit (Agilent Technologies). The genomic DNA was then sheared into 10- to 20-kb fragments using a g-TUBE device (Covaris). The sequencing library was constructed using the SMRTbell Express template prep kit v.2.0 (PacBio) following the manufacturer’s instructions and was sequenced using the PacBio Sequel IIe system. To generate high-fidelity (HiFi) reads, adapter trimming and computation of circular consensus sequencing were performed via SMRT Link v.11.0.0.146107. Consensus sequences with an average quality value of <20 were excluded, resulting in 17,329 HiFi reads with a mean size of 10,541 bp. After excluding HiFi reads shorter than 1,000 bp using Fitlong v.0.2.0 (https://github.com/rrwick/Filtlong), the remaining 15,064 reads were used for *de novo* assembly with Flye v.2.9 (4). Genomic circularity was confirmed by assessing the assembly graph using Bandage v.0.8.1 (https://github.com/rrwick/Bandage) (5). The assembled genomic data integrity was confirmed using CheckM v.1.2.0 (https://github.com/Ecogenomics/CheckM) (6), showing 99.5% completeness and 0.93% contamination. This resulted in a single 7,071,873-bp contig, specified as circular using Flye, with a coverage of 26× and an overall G + C content of 67.4%.

Functional annotation of the genome was performed using Prokka v.1.14.5 (https://github.com/tseemann/prokka) (7), predicted 6,510 genes, including 6,428 codings, 10 rRNA, and 70 tRNA. *In silico* DNA-DNA hybridization (DDH) value was calculated using the Genome-to-Genome Distance Calculator v.3.0 (https://ggdc.dsmz.de) (8), and average nucleotide identity (ANI) value was calculated using the ANI calculator (https://www.ezbiocloud.net/tools/ani) (9). The results showed a DDH value of 70.7% and an ANI value of 96.7% toward *A. xylosoxidans* LMG 1863 (assembly accession number GCA_000508285). Default parameters were used for all software unless otherwise specified.

## Data Availability

The genome sequence of *Achromobacter* xylosoxidans strain H1_3_1 has been deposited at the DNA Data Bank of Japan (DDBJ) under the accession number AP028040. The raw reads have been deposited in the DDBJ Sequence Read Archive under accession number DRA016141. The BioSample and BioProject accession numbers are SAMD00597894 and PRJDB15726, respectively.

## References

[1] Talbot NP , Flight WG . 2016. Severe Achromobacter xylosoxidans infection and loss of sputum bacterial diversity in an adult patient with cystic fibrosis. Paediatr Respir Rev 20 Suppl:27–29. doi:10.1016/j.prrv.2016.06.011 27374622

[2] Busse HJ , Auling G . 2015. Achromobacter, p 1–14. In Bergey’s manual of systematics of archaea and bacteria. doi:10.1002/9781118960608

[3] Feng X , He S , Sato T , Kondo T , Uema K , Sato K , Kobayashi H . 2023. Enrichment of hydrogen-oxidizing bacteria using a hybrid biological-inorganic system. J Biosci Bioeng 135:250–257. doi:10.1016/j.jbiosc.2022.12.011 36650080

[4] Kolmogorov M , Yuan J , Lin Y , Pevzner PA . 2019. Assembly of long, error-prone reads using repeat graphs. Nat Biotechnol 37:540–546. doi:10.1038/s41587-019-0072-8 30936562

[5] Wick RR , Schultz MB , Zobel J , Holt KE . 2015. Bandage: interactive visualization of de novo genome assemblies. Bioinformatics 31:3350–3352. doi:10.1093/bioinformatics/btv383 26099265 PMC4595904

[6] Parks DH , Imelfort M , Skennerton CT , Hugenholtz P , Tyson GW . 2015. CheckM: assessing the quality of microbial genomes recovered from isolates, single cells, and metagenomes. Genome Res 25:1043–1055. doi:10.1101/gr.186072.114 25977477 PMC4484387

[7] Seemann T . 2014. Prokka: rapid prokaryotic genome annotation. Bioinformatics 30:2068–2069. doi:10.1093/bioinformatics/btu153 24642063

[8] Meier-Kolthoff JP , Carbasse JS , Peinado-Olarte RL , Göker M . 2022. TYGS and LPSN: a database tandem for fast and reliable genome-based classification and nomenclature of prokaryotes. Nucleic Acids Res 50:D801–D807. doi:10.1093/nar/gkab902 34634793 PMC8728197

[9] Yoon SH , Ha SM , Lim J , Kwon S , Chun J . 2017. A large-scale evaluation of algorithms to calculate average nucleotide identity. Antonie Van Leeuwenhoek 110:1281–1286. doi:10.1007/s10482-017-0844-4 28204908

